# Supplementary material for: A C. elegans Zona Pellucida domain protein functions via its ZPc domain
Source: PLoS Genet. 2020 Nov 3;16(11):e1009188. doi: 10.1371/journal.pgen.1009188 (PMC7665627; doi:10.1371/journal.pgen.1009188)
Supplement: S1 Fig — A-A’) Levels of LET-653 fluorescence in the 1.5 fold embryo. Functional transgenes were generally expressed at similar levels to endogenous LET-653 in the duct lumen, but accumulated to much higher levels in the extraembryonic space. Error bars; standard error. B) Quantification of membrane-associated vs. luminal LET-653 in the mid-L4 vulva for all groups. C-terminally and N-terminally tagged LET-653(ZP) and LET-653(ZPc) are not significantly different from one another. However, LET-653(ZPc, AYAA) is significantly more enriched at the apical membrane than LET-653(ZP, AYAA), and this result correlates with increased function in LET-653(ZPc, AYAA) vs LET-653(ZP, AYAA) (Figs 4–5). Dashed line indicates a ratio of 1, where membrane fluorescence is equal to luminal fluorescence. Error bars; standard error. (DOCX) [file pgen.1009188.s001.docx]

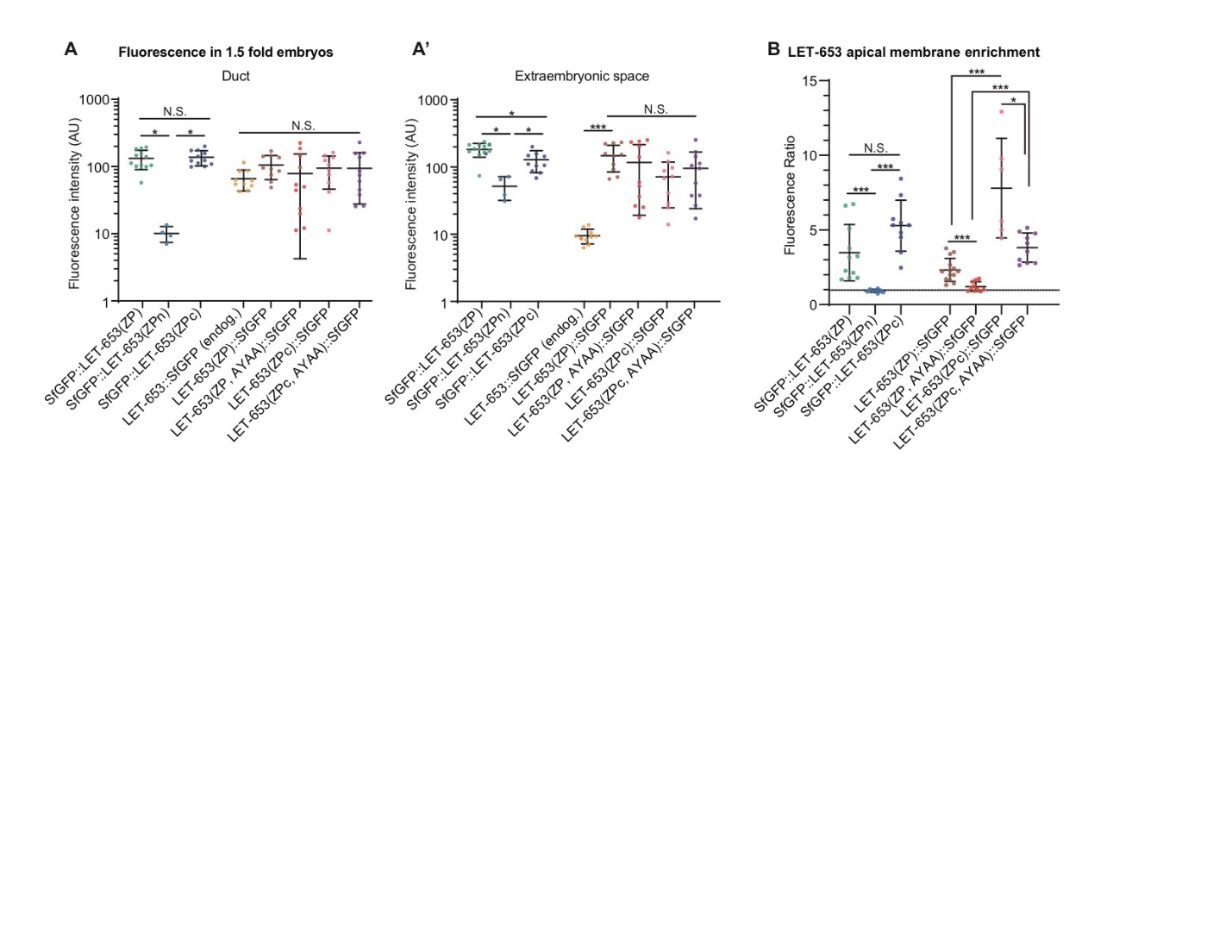


S1 Fig. Method of measuring LET-653 in embryos and vulva

A-A’) Levels of LET-653 fluorescence in the 1.5 fold embryo. Functional transgenes were generally expressed at similar levels to endogenous LET-653 in the duct lumen, but accumulated to much higher levels in the extraembryonic space. Error bars; standard error. B) Quantification of membrane-associated vs. luminal LET-653 in the mid-L4 vulva for all groups. C-terminally and N-terminally tagged LET-653(ZP) and LET-653(ZPc) are not significantly different from one another. However, LET-653(ZPc, AYAA) is significantly more enriched at the apical membrane than LET-653(ZP, AYAA), and this result correlates with increased function in LET-653(ZPc, AYAA) vs LET-653(ZP, AYAA) (Figures 4-5). Dashed line indicates a ratio of 1, where membrane fluorescence is equal to luminal fluorescence. Error bars; standard error.
