# Supplementary material for: A C. elegans Zona Pellucida domain protein functions via its ZPc domain
Source: PLoS Genet. 2020 Nov 3;16(11):e1009188. doi: 10.1371/journal.pgen.1009188 (PMC7665627; doi:10.1371/journal.pgen.1009188)
Supplement: S2 Fig — LET-653(ZP) and LET-653(ZP, AYAA) are both present in the duct lumen at 1.5 fold stage but are gradually cleared over several hours. Presence of LET-653 in the duct lumen was assessed visually via epifluorescence microscopy. LET-653(ZP, AYAA) was sometimes too faint to be detected within the duct lumen at the 1.5 fold stage. n = 20 for each time point. (DOCX) [file pgen.1009188.s002.docx]

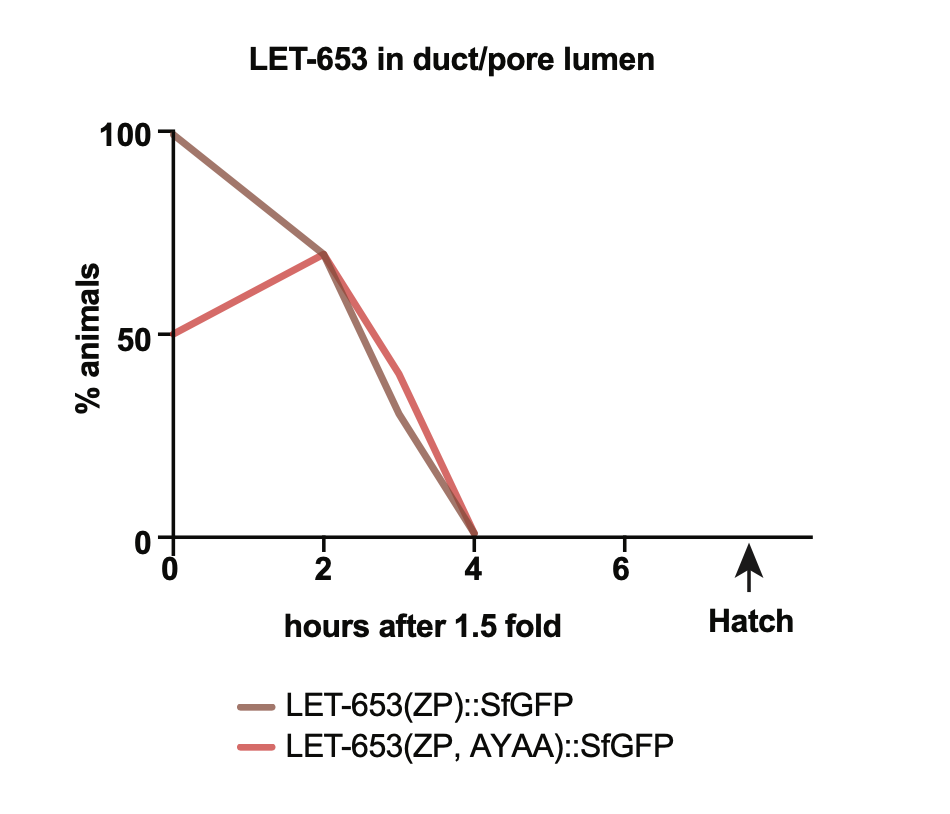


S2 Fig. LET-653(ZP, AYAA) is cleared normally from the embryonic duct.

LET-653(ZP) and LET-653(ZP, AYAA) are both present in the duct lumen at 1.5 fold stage but are gradually cleared over several hours. Presence of LET-653 in the duct lumen was assessed visually via epifluorescence microscopy. LET-653(ZP, AYAA) was sometimes too faint to be detected within the duct lumen at the 1.5 fold stage. n = 20 for each time point.
