# Supplementary material for: A C. elegans Zona Pellucida domain protein functions via its ZPc domain
Source: PLoS Genet. 2020 Nov 3;16(11):e1009188. doi: 10.1371/journal.pgen.1009188 (PMC7665627; doi:10.1371/journal.pgen.1009188)
Supplement: S3 Fig — Fluorescence recovery after photobleaching (FRAP) of LET-653 translational fusions in the vulva lumen. These experiments, which used integrated transgenes, gave similar results to those in Fig 6. Pre-bleach, bleach, and post-bleach frames taken from FRAP experiment on mid-L4 vulvas. Red box; bleached region of interest. Black box; unbleached region of interest. Background regions of interest are outside the field of view. A) FRAP of SfGFP (csEx636). Bleaching is not detected, possibly due to rapid replacement of bleached molecules by unbleached neighbors. Representative of n = 6 replicates. B) FRAP of SfGFP::LET-653(ZP) (csIs66). Representative of n = 6 replicates. C) FRAP of SfGFP::LET-653(ZPc) (csIs92). Representative of n = 6 replicates. D) Fluorescence recovery curves with mean and standard error for each LET-653 fusion protein. t = 0s represents the first post-bleach frame. The limited SfGFP bleaching meant that mobile fractions were often negative values and could not be reliably calculated. E) Mobile fraction calculated from recovery curves. No significant difference was detected between groups, Mann Whitney two-tailed U test. (DOCX) [file pgen.1009188.s003.docx]

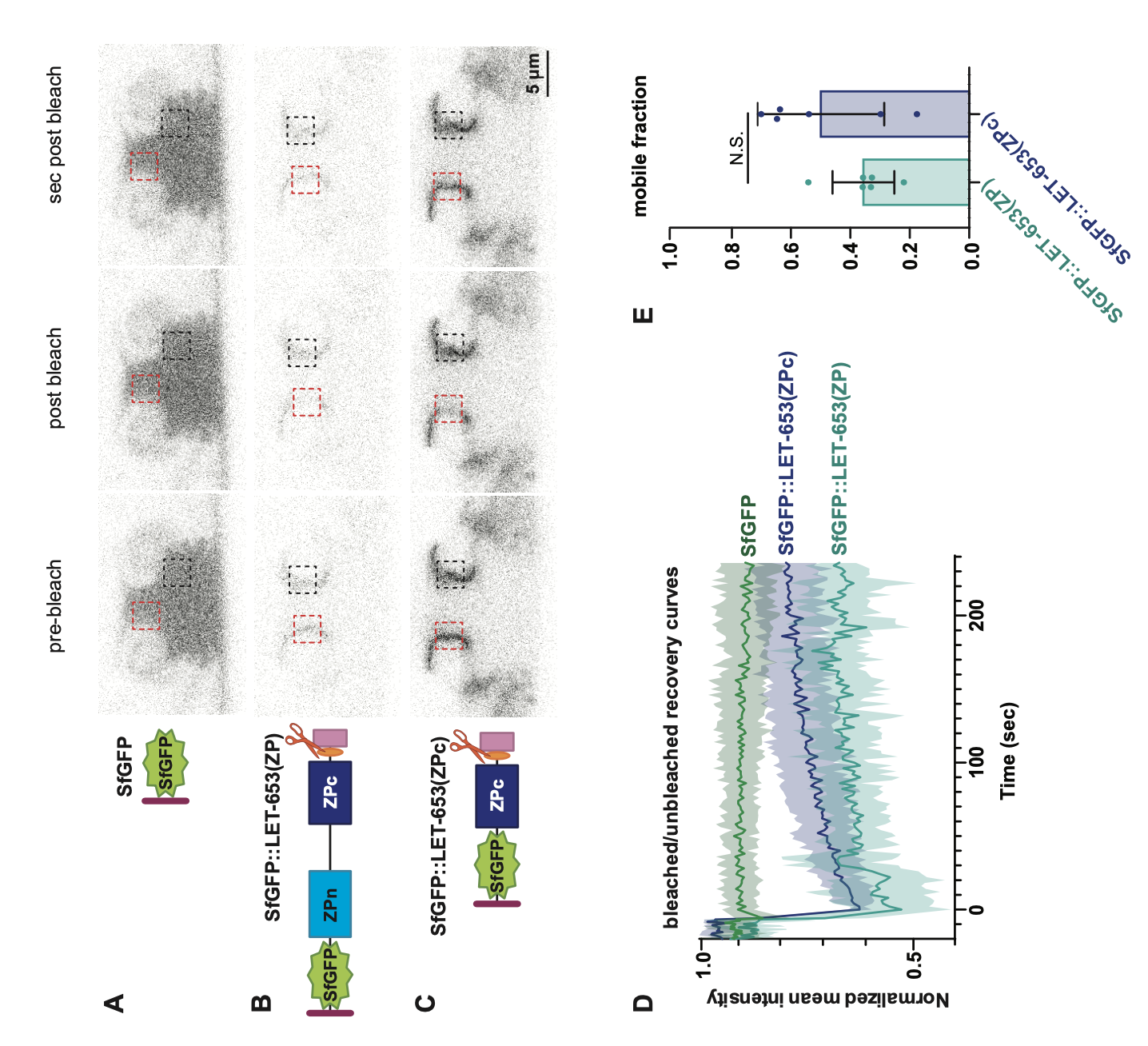


S3 Fig. LET-653(ZPc) and LET-653(ZP) show similarly limited mobility

Fluorescence recovery after photobleaching (FRAP) of LET-653 translational fusions in the vulva lumen. These experiments, which used integrated transgenes, gave similar results to those in Figure 6. Pre-bleach, bleach, and post-bleach frames taken from FRAP experiment on mid-L4 vulvas. Red box; bleached region of interest. Black box; unbleached region of interest. Background regions of interest are outside the field of view. A) FRAP of SfGFP (*csEx636*). Bleaching is not detected, possibly due to rapid replacement of bleached molecules by unbleached neighbors. Representative of n = 6 replicates. B) FRAP of SfGFP::LET-653(ZP) (*csIs66*). Representative of n = 6 replicates. C) FRAP of SfGFP::LET-653(ZPc) (*csIs92*). Representative of n = 6 replicates. D) Fluorescence recovery curves with mean and standard error for each LET-653 fusion protein. t = 0s represents the first post-bleach frame. The limited SfGFP bleaching meant that mobile fractions were often negative values and could not be reliably calculated. E) Mobile fraction calculated from recovery curves. No significant difference was detected between groups, Mann Whitney two-tailed U test.
