# Supplementary material for: A C. elegans Zona Pellucida domain protein functions via its ZPc domain
Source: PLoS Genet. 2020 Nov 3;16(11):e1009188. doi: 10.1371/journal.pgen.1009188 (PMC7665627; doi:10.1371/journal.pgen.1009188)
Supplement: S4 Fig — S2R+ cells were transfected with 1 μg DNA of the indicated constructs. A-F) Epifluorescent images of cells 72 hrs post-transfection, representative of >100 cells per condition. Large intracellular or cell-bound fibrils (arrows) were visible from SfGFP::DYF-7 [54] (B), but not from SfGFP alone or any LET-653 protein fusion. Black bar; transmembrane domain. Purple boxes; DYF-7 ZP domain. G) Western blot of S2R+ cell lysate probed with anti-GFP. Both un-cleaved and cleaved versions (arrowheads) of LET-653(ZP) and LET-653(ZPc) were visible. H) Western blot of S2R+ cell media probed with anti-GFP. LET-653(ZP) and LET-653(ZPc) were efficiently cleaved and cleavage products were visible in the media (arrowheads). LET-653(ZP, AYAA) was not cleaved but was still secreted into the media. EV; Empty vector. Western blot of media is representative of three blots from the three independent transfection experiments. Western blot of cell lysates was performed using cells from transfection 2. All images shown are of cells or media from transfection 2. (DOCX) [file pgen.1009188.s004.docx]

S4 Fig. LET-653 is cleaved when expressed from S2R+ cells

S2R+ cells were transfected with 1 µg DNA of the indicated constructs. A-F) Epifluorescent images of cells 72 hrs post-transfection, representative of >100 cells per condition. Large intracellular or cell-bound fibrils (arrows) were visible from SfGFP::DYF-7 [54] (B), but not from SfGFP alone or any LET-653 protein fusion. Black bar; transmembrane domain. Purple boxes; DYF-7 ZP domain. G) Western blot of S2R+ cell lysate probed with anti-GFP. Both un-cleaved and cleaved versions (arrowheads) of LET-653(ZP) and LET-653(ZPc) were visible. H) Western blot of S2R+ cell media probed with anti-GFP. LET-653(ZP) and LET-653(ZPc) were efficiently cleaved and cleavage products were visible in the media (arrowheads). LET-653(ZP, AYAA) was not cleaved but was still secreted into the media. EV; Empty vector. Western blot of media is representative of three blots from the three independent transfection experiments. Western blot of cell lysates was performed using cells from transfection 2. All images shown are of cells or media from transfection 2.
