# Supplementary material for: A C. elegans Zona Pellucida domain protein functions via its ZPc domain
Source: PLoS Genet. 2020 Nov 3;16(11):e1009188. doi: 10.1371/journal.pgen.1009188 (PMC7665627; doi:10.1371/journal.pgen.1009188)
Supplement: S5 Fig — Quantification of aggregate density in media thawed and mounted on glass slides. A) Aggregate density in media from transfection 2, shown in Fig 7. *p = 0.0079, two-tailed Mann Whitney U test. B) Aggregate density in media from transfection 1. Aggregate number was high across all genotypes. LET-653(ZP,AYAA) had significantly more aggregates than the other groups. *p = 0.0079, two-tailed Mann Whitney U test. Error bars; standard error. (DOCX) [file pgen.1009188.s005.docx]

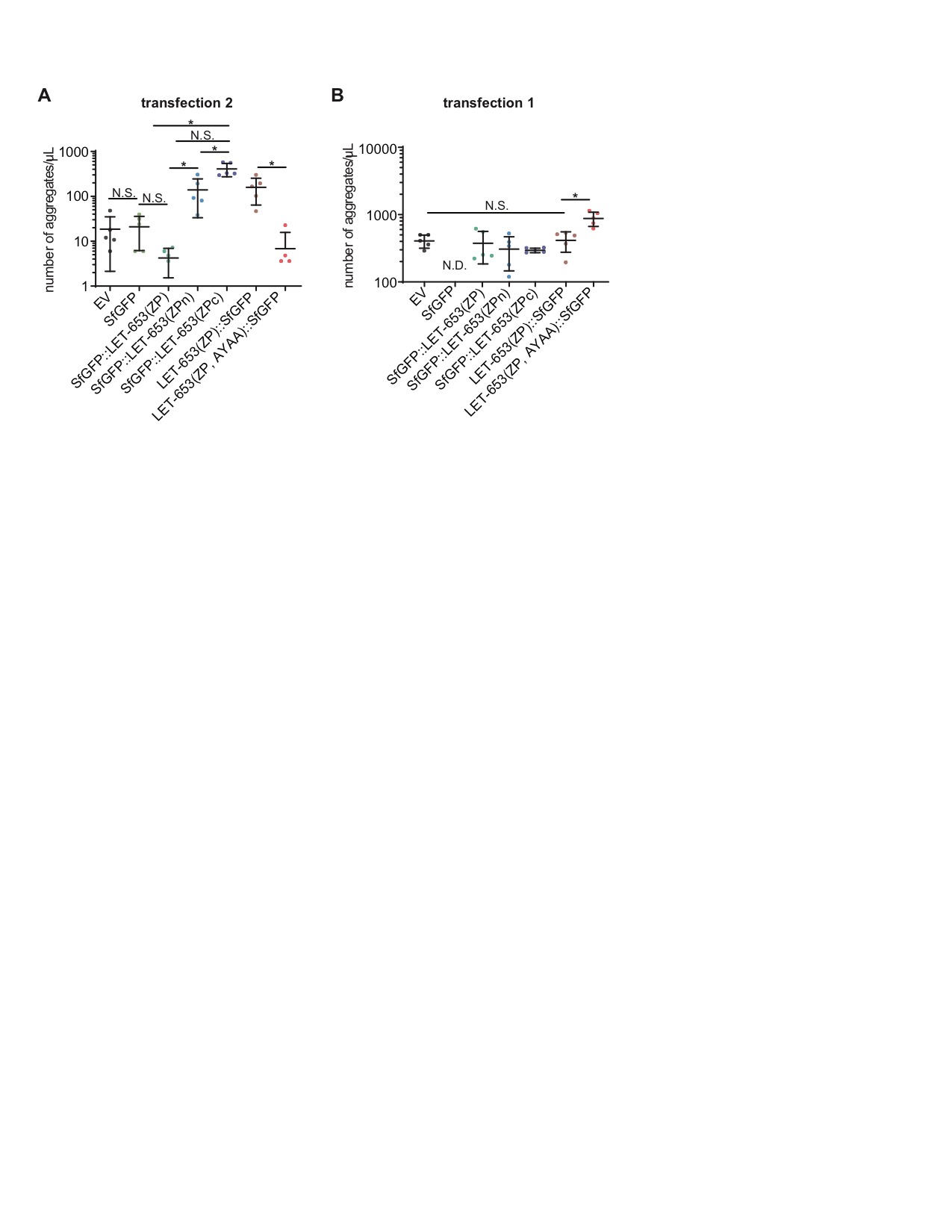
 S5 Fig. Aggregate number does not correlate with LET-653 function

Quantification of aggregate density in media thawed and mounted on glass slides. A) Aggregate density in media from transfection 2, shown in Figure 7. *p=0.0079, two-tailed Mann Whitney U test. B) Aggregate density in media from transfection 1. Aggregate number was high across all genotypes. LET-653(ZP,AYAA) had significantly more aggregates than the other groups. *p=0.0079, two-tailed Mann Whitney U test. Error bars; standard error.
