# Supplementary material for: A C. elegans Zona Pellucida domain protein functions via its ZPc domain
Source: PLoS Genet. 2020 Nov 3;16(11):e1009188. doi: 10.1371/journal.pgen.1009188 (PMC7665627; doi:10.1371/journal.pgen.1009188)
Supplement: S6 Fig — Alignment of ZPn domains based on Monne et al., 2008[41] and Gill et al., 2016 [52]. Grey shading corresponds to known beta-strands in ZP3, blue indicates conserved cysteines and yellow indicates conserved aromatic residues. Like LET-653, Oit3 lacks the conserved aromatic residue in the F strand between Cys3 and Cys4, and DMBT1 lacks the second conserved aromatic residue after Cys4. These two residues interact in the ZP3-N crystal structure [41] and are thought to be critical for ZPn polymerization. Genbank accession numbers: Ce_LET-653b: NP_001021337; Ce_DYF-7: NP_509630; Dm_Dpy: NP_001245875; Hs_UMOD: NP_003352; Hs_TectA: NP_005413; Hs_ZP3: NP_001103824; Hs_DMBT1: NP_004397; Hs_Oit3: NP_689848; Hs_Eng: NP_001108225. (DOCX) [file pgen.1009188.s006.docx]

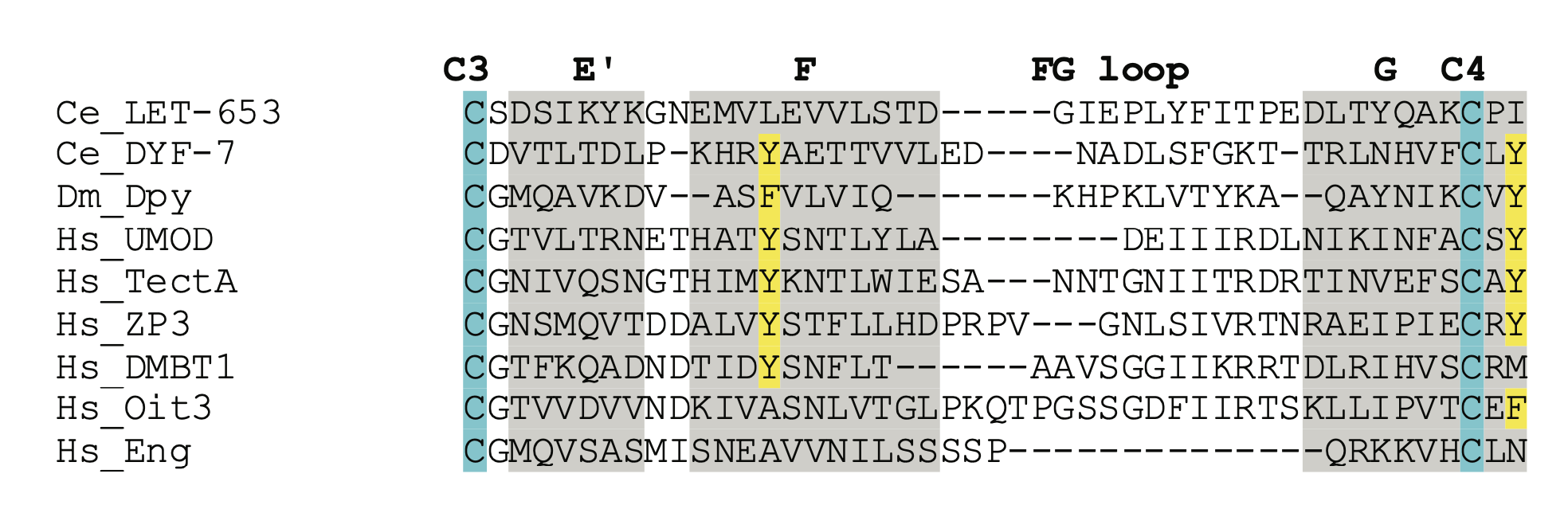


S6 Fig. The human ZP proteins Oit3 and DMBT1 lack key tyrosine residues

Alignment of ZPn domains based on Monne et al., 2008 [41] and Gill et al., 2016 [52]. Grey shading corresponds to known beta-strands in ZP3, blue indicates conserved cysteines and yellow indicates conserved aromatic residues. Like LET-653, Oit3 lacks the conserved aromatic residue in the F strand between Cys3 and Cys4, and DMBT1 lacks the second conserved aromatic residue after Cys4. These two residues interact in the ZP3-N crystal structure [41] and are thought to be critical for ZPn polymerization. Genbank accession numbers: Ce_LET-653b: NP_001021337; Ce_DYF-7: NP_509630; Dm_Dpy: NP_001245875; Hs_UMOD: NP_003352 ; Hs_TectA: NP_005413; Hs_ZP3: NP_001103824; Hs_DMBT1: NP_004397; Hs_Oit3: NP_689848; Hs_Eng: NP_001108225.
