# Supplementary material for: A C. elegans Zona Pellucida domain protein functions via its ZPc domain
Source: PLoS Genet. 2020 Nov 3;16(11):e1009188. doi: 10.1371/journal.pgen.1009188 (PMC7665627; doi:10.1371/journal.pgen.1009188)
Supplement: S1 Table — All strains used or generated in this study are listed with full genotypes. (DOCX) [file pgen.1009188.s007.docx]

S1 Table. Strains generated in this study.

| **Strain** | **Genotype** | **Citations** |
| --- | --- | --- |
| UP3121 | *csEx624 [let-653pro::SfGFP::LET-653(ZP); lin48pro::mRFP]* | [52] |
| UP3244 | *let-653(cs178) IV; csEx358 [lpr-1pro::LET-653b; unc-119pro::GFP]* | [52] |
| UP3342 | *let-653(cs178) IV; csEx766 [lin48pro::LET-653b::SfGFP;*  *myo-2pro::GFP]* | [51] |
| UP3422 | *csIs66 [let-653pro::LET-653(ZP)::SfGFP;*  *let-653pro::PH::mCherry] X* | [51] |
| UP3432 | *let-653(cs178) IV; csEx821 [let-653pro::SfGFP::LET-653(ZPc);*  *lin-48pro::mRFP]* |  |
| UP3448 | *csEx822 [let-653pro::SfGFP::LET-653(ZPc); lin-48pro::mRFP]* |  |
| UP3449 | *csEx821 [let-653pro::SfGFP::LET-653(ZPc); lin-48pro::mRFP]* |  |
| UP3462 | *let-653(cs178) IV; csIs66 [let-653pro::LET-653(ZP)::SfGFP;*  *let-653pro::PH::mCherry] X* |  |
| UP3465 | *let-653(cs178) IV; csEx828 [let-653pro::LET-653(ZPc);*  *lin-48pro::mRFP]* |  |
| UP3466 | *let-653(cs178) IV; csEx829 [let-653pro::LET-653(ZPc);*  *lin-48pro::mRFP]* |  |
| UP3514 | *csEx841 [let-653pro::LET-653(ZPc)::SfGFP; lin-48pro::mRFP]* |  |
| UP3515 | *csEx842 [let-653pro::LET-653(ZPc)::SfGFP; lin-48pro::mRFP]* |  |
| UP3516 | *csEx843 [let-653pro::SfGFP::LET-653(ZPn); lin-48pro::mRFP]* |  |
| UP3517 | *csEx844 [let-653pro::SfGFP::LET-653(ZPn); lin-48pro::mRFP]* |  |
| UP3594 | *let-653(cs178) IV; csEx841 [let-653pro::LET-653(ZPc)::SfGFP;*  *lin-48pro:mRFP]* |  |
| UP3630 | *csEx882 [let-653pro::LET-653(ZP,AYAA)::SfGFP;*  *lin-48pro::mRFP]* |  |
| UP3630 | *csEx882 [let-653pro::LET-653(ZP,AYAA)::SfGFP;*  *lin-48pro::mRFP]* |  |
| UP3631 | *csEx883 [let-653pro::LET-653(ZP,AYAA)::SfGFP;*  *lin-48pro::mRFP]* |  |
| UP3746 | *let-653(cs262 [LET-653::SfGFP]) IV* | [61] |
| UP3777 | *let-653(cs178) IV; csEx889 [let-653pro::SfGFP::LET-653(ZPn);*  *lin-48pro::mRFP]; csEx358* |  |
| UP3806 | *let-653(cs178) IV; csEx885 [let-653pro::LET-653(ZP,AYAA);*  *lin-48pro::mRFP]* |  |
| UP3807 | *let-653(cs178) IV; csEx893 [let-653pro::SfGFP::LET-653(ZPn);*  *lin-48pro::mRFP]; csEx358* |  |
| UP3843 | *csIs91 [let-653pro::SfGFP::LET-653(ZPc); lin-48pro::mRFP]* |  |
| UP3844 | *csIs92 [let-653pro::SfGFP::LET-653(ZPc); lin-48pro::mRFP]* |  |
| UP3845 | *csIs93 [let-653pro::SfGFP::LET-653(ZPc); lin-48pro::mRFP]* |  |
| UP3846 | *csIs94 [let-653pro::LET-653(ZP, AYAA)::SfGFP; lin-48pro::mRFP]* |  |
| UP3847 | *csIs95 [let-653pro::LET-653(ZP, AYAA)::SfGFP; lin-48pro::mRFP]* |  |
| UP3848 | *csEx905 [let-653pro::LET-653(ZP)::SfGFP; lin-48pro::mRFP]* |  |
| UP3849 | *csIs96 [let-653pro::LET-653(ZP)::SfGFP; lin-48pro::mRFP]* |  |
| UP3865 | *csEx913 [let-653pro::SfGFP::LET-653(ZPc-½Cterm);*  *lin-48pro::mRFP]* |  |
| UP3866 | *csEx916 [let-653pro::LET-653(ZPn+Cterm)::SfGFP;*  *lin-48pro::mRFP]* |  |
| UP3867 | *csEx912 [let-653pro::SfGFP::LET-653(ZPc-½Cterm);*  *lin-48pro::mRFP]* |  |
| UP3868 | *csEx911 [let-653pro::SfGFP::LET-653(ZPc-½Cterm);*  *lin-48pro::mRFP]* |  |
| UP3869 | *csEx910 [let-653pro::SfGFP::LET-653(ZPc-½Cterm);*  *lin-48pro::mRFP]* |  |
| UP3870 | *csEx917 [let-653pro::LET-653(ZPn+Cterm)::SfGFP;*  *lin-48pro::mRFP]* |  |
| UP3871 | *csEx915 [let-653pro::LET-653(ZPn+Cterm)::SfGFP;*  *lin-48pro::mRFP]* |  |
| UP3872 | *csEx914 [let-653pro::LET-653(ZPn+Cterm)::SfGFP;*  *lin-48pro::mRFP]* |  |
| UP3880 | *let-653(cs178) IV; csEx696 [let-653pro::LET-653(ZP)::SfGFP;*  *lin-48pro::mRFP]* |  |
| UP3919 | *let-653(cs178) IV; csEx913 [let-653pro::SfGFP::*  *LET-653(ZPc-½Cterm); lin-48pro::mRFP]; csEx766* |  |
| UP3929 | *let-653(cs178) IV; csEx915 [let-653pro::*  *LET-653(ZPn+Cterm)::SfGFP; lin-48pro::mRFP]; csEx358* |  |
| UP3930 | *let-653(cs178) IV; csEx917 [let-653pro::*  *LET-653(ZPn+Cterm)::SfGFP; lin-48pro::mRFP]; csEx358* |  |
| UP3931 | *let-653(cs178) IV; csEx915 [let-653pro::*  *LET-653(ZPn+Cterm)::SfGFP; lin-48pro::mRFP]; csEx358* |  |
| UP3951 | *let-653(cs178) IV; csEx911 [let-653pro::SfGFP::*  *LET-653(ZPc-½Cterm); lin-48pro::mRFP]; csEx766* |  |
| UP3952 | *let-653(cs178) IV; csEx886 [let-653pro::*  *LET-653(ZP,AYAA)::SfGFP; lin-48pro::mRFP]; csEx358* |  |
| UP3953 | *let-653(cs178) IV; csIs96 [let-653pro::LET-653(ZP)::SfGFP;*  *lin-48pro::mRFP]* |  |
| UP3988 | *csEx934 [let-653pro::LET-653(ZPc,AYAA)::SfGFP;*  *lin-48pro::mRFP]* |  |
| UP3997 | *csEx935 [let-653pro::LET-653(ZPc,AYAA)::SfGFP;*  *lin-48pro::mRFP]* |  |
| UP3998 | *csEx936 [let-653pro::LET-653(ZPc,AYAA)::SfGFP;*  *lin-48pro::mRFP]* |  |
| UP3999 | *let-653(cs178) IV; csEx938 [let-653pro::*  *LET-653(ZPc,AYAA)::SfGFP; lin-48pro::mRFP]; csEx358* |  |
| UP4000 | *let-653(cs178) IV; csEx937 [let-653pro::*  *LET-653(ZPc,AYAA)::SfGFP; lin-48pro::mRFP]; csEx358* |  |
