# Supplementary material for: A C. elegans Zona Pellucida domain protein functions via its ZPc domain
Source: PLoS Genet. 2020 Nov 3;16(11):e1009188. doi: 10.1371/journal.pgen.1009188 (PMC7665627; doi:10.1371/journal.pgen.1009188)
Supplement: S2 Table — All plasmids generated in this study are listed with their contents and mechanism of assembly. (DOCX) [file pgen.1009188.s008.docx]

**S2 Table. Plasmids generated in this study**

| **Plasmid** | **Contains** | **Transgenes** | **Construction** |
| --- | --- | --- | --- |
| pJC30 | *let-653pro::*  *SfGFP::*  *LET-653(ZPc)* | csEx821 | LET-653(ZPc) was PCR amplified from pJAF7 [52] using primers oJC77 (GGGAAAGCTAGCCATGTAGGAGCTCCCGCATCT) and oCP2 (GGGGGTACCTCAGATGTTTCCAGTTCGAAC) and inserted into pJAF5 as a NheI – KpnI fragment. ssSfGFP was cut from pRFR21 [52] and inserted upstream as a NheI fragment. |
| pJC37 | *let-653pro::*  *LET-653(ZPc)::*  *SfGFP* | csEx841 | LET-653(ZPc+Cterm)::SfGFP was amplified from pJAF7 using primers oJC116 (CCCGGGGCTAGCATGCGACATCCACTAATTTCTCTACTATTGCTAATAGCATTCTACTCTACATCGTCAGAAGCACATGTAGGAGCTCCCGCATC) and oJC144 (GGGTTTCTTAAGGCCGCCTGATGCGG) and inserted into pJAF5 as a NheI – AflII fragment. SfGFP was added as a KpnI fragment. |
| pJC38 | *let-653pro::*  *LET-653(ZPc)* | csEx828, csEx829 | LET-653(ZPc+Cterm) was amplified from pJAF7 using primers oJC116 (CCCGGGGCTAGCA  TGCGACATCCACTAATTTCTCTACTATTGCTAATAGCATTCTACTCTACATCGTCAGAAGCACATGTAGGAGCTCCCGCATC) and oJC144 (GGGTTTCTTAAGGCCGC  CTGATGCGG) and inserted into pJAF5 as a NheI – AflII fragment. |
| pJC42 | *let-653pro:: SfGFP::*  *LET-653(ZPn)* | csEx889, csEx893 | ssSfGFP::LET-653(ZPn) was PCR amplified from pRFR21 [52] using primers oJC132 (CCCAAACCCGGGATTGGCCAAAGGACC) and oJC133 (GGGTTTGATATCTCAAGTGGTCGTTG  TTTGTGGTG) and inserted into pJAF5 [52] as a XmaI – EcoRV fragment |
| pJC53 | *let-653pro::*  *LET-653(ZP, AYAA)::*  *SfGFP* | csEx882, csEx885, csEx886 | LET-653(ZP, AYAA)::SfGFP was amplified from pJC21 using primers oJC206 (CCCAAAATG  CATGCTTACGCGGCAGAATTATC) and oJC144 (GGGTTTCTTAAGGCCGCCTGATGCGG) and inserted into pJC21 as a NsiI – AflII fragment. |
| pJC93 | *let-653pro::*  *SfGFP::*  *LET-653*  *(ZPc-1/2Cterm)* | csEx911, csEx913 | LET-653(ZPc+EHP) was amplified from pJC30 using primers oJC77 (GGGAAAGCTAGCCA  TGTAGGAGCTCCCGCATCT) and oJC390 (GGGAAA  GGTACCCTATTTAGATTTCGACGGATTTC) as a NheI – KpnI fragment. ssSfGFP was cut from pRFR21 and inserted as a NheI fragment. |
| pJC94 | *let-653pro::*  *LET-653*  *(ZPn+Cterm)::*  *SfGFP* | csEx915, csEx917 | LET-653(ZPn) and LET-653(Cterm)::SfGFP were independently amplified from pJC21 using primers oCP10 (GGGGCTAGCAAAATGCGAC  ATCCACTAATTTCTCTAC), oJC394 (GGGAAATGCG  GCCGCAGTGGTCGTTGTTTGTGGTG), oJC395 (GGG  AAAGCGGCCGCAAGCTACATGCATCGATACAGG), and oJC396 (GGGAAAGATATCTCATTTGTAGAGCTCATC  CATG) and PCR sewn together before being inserted into pJAF5 [52] as a NheI – EcoRV fragment. |
| pJC95 | *pAC5.1::*  *SfGFP* |  | SfGFP with signal sequence amplified from pJC42, PCR sewn with *unc-54* 3'UTR from pCW14 [54], and inserted into pAC5.1 as XbaI-ApaI fragment. |
| pJC96 | *pAC5.1::*  *SfGFP::*  *LET-653(ZPn)* |  | SfGFP::LET-653(ZPn) amplified from pJC42, PCR sewn with *unc-54* 3'UTR from pCW14 [54], and inserted into pAC5.1 as XbaI-ApaI fragment. |
| pJC97 | *pAC5.1::*  *SfGFP::*  *LET-653(ZPc)* |  | SfGFP::LET-653(ZPc) amplified from pJC30, PCR sewn with *unc-54* 3'UTR from pCW14 [54], and inserted into pAC5.1 as XbaI-ApaI fragment. |
| pJC98 | *pAC5.1::*  *SfGFP::*  *LET-653(ZP)* |  | SfGFP::LET-653(ZP) amplified from pRFR21 [52], PCR sewn with *unc-54* 3'UTR from pCW14 [54], and inserted into pAC5.1 as XbaI-ApaI fragment. |
| pJC99 | *pAC5.1::LET-653(ZP)::*  *SfGFP* |  | LET-653(ZP)::SfGFP amplified from pJC21, PCR sewn with *unc-54* 3'UTR from pCW14 [54], and inserted into pAC5.1 as XbaI-ApaI fragment. |
| pJC100 | *pAC5.1::*  *LET-653(ZP, AYAA)::SfGFP* |  | LET-653(ZP, AYAA)::SfGFP amplified from pJC53, PCR sewn with *unc-54* 3'UTR from pCW14 [54], and inserted into pAC5.1 as XbaI-ApaI fragment. |
| pJC103 | *let-653pro::*  *LET-653*  *(ZPc, AYAA)::*  *SfGFP* | csEx934, csEx937, csEx938 | LET-653(ZPc, AYAA)::SfGFP was amplified from pJC21 using primers oJC206 (CCCAAAATGCATGCTTACGCGGCAGAATTATC) and oJC144 (GGGTTTCTTAAGGCCGCCTGATGCGG) and inserted into pJC37 as a NsiI – AflII fragment. |
